# Notes

**Published:** 1895-09

**Authors:** 


### Notes.

The greatest disappointments in progressive medicine within the last decade have originated in Germany and France.—*Medicine.*

Professor Hare says, in cases of fistula a cure can often be effected without operation by touching up the edges of the fistulous tract with nitric acid, which causes an outpouring of granulation tissue and thus plugs up the openings.

On April 30th, the Massachusetts House of Representatives, by a vote of 149 to 23, refused to order to a third reading the bill to prohibit insurance of the lives of children under ten years of age, thus ending the matter for the present session.

Dr. Frank Parsons Norbury who recently removed to St. Louis to assume the editorial management of the *Medical Fortnightly*, has been elected to the chair of Practice of Medicine and Clinical Medicine in the St. Louis College of Physicians and Surgeons.

An interesting comparison has been made between the number of physicians practicing in civilized and uncivilized countries. Thus New York City has 3,500 physicians for her two millions. China has twelve hospitals, and twenty-five physicians for 400,000,000.

A Canadian newspaper calls attention to a nursing bottle advertisement, which concludes with the words: "When the baby is done drinking it must be unscrewed and laid in a cool place under a tap. If the baby does not thrive on fresh milk, it should be boiled." Poor baby!

Laboratory inoculation has had another example in the case of Dr. Ruffer, of London. He was carrying on investigations concerning antitoxin, and accidentally inoculated himself with diphtheria. He was treated with serum, and after a tedious illness, is recovered.—*Columbus Medical Journal.*

Dr. S. Weir Mitchell, the eminent neurologist of Philadelphia, already a possessor of the degree of LL. D., had the same conferred on him, August 1st, by the University of Edinburgh. In the University oration he was described as "the chief ornament of medical science in the New World."—*Columbus Medical Journal.*

The promoters of a German erysipelas serum for cancer treatment have adopted the proprietary name "Anticancrin" for their product. Drs. Emmerich and Scholl could have saved themselves this trouble; clinical reports, including the records of their own tests, show that their serum possesses no special value—and, anyway, we will not need their serum in this country, as we have long had it available from domestic laboratories.

The death of Dr. Alfred L. Loomis has led to a number of changes in the Faculty of the Medical Department of the University of the City of New York. Dr. Wm. H. Thomson is now Professor of Medicine; Dr. W. Gilman Thompson, Professor of Materia Medica, Therapeutics and Clinical Medicine; Dr. Charles L. Bristol, Lecturer on Physiology; Dr. Henry P. Loomis, Professor of Pathology, and Dr. Charles E. Quimby, Adjunct Professor of Practice of Medicine.

AN ORDER OF THE COURT OF INTEREST TO DR. KEELEY.—According to the *Chicago Herald* of May 2d, Judge Myers, of the United States District Court, in session at Leavenworth, Kan., has made a very important order affecting the rights of Dr. Leslie E. Keeley. W. F. Johnson, of Topeka, sues Dr. Keeley for $100,000 damages, the petition reciting that plaintiff has been made a physical wreck because of the gold cure. Judge Myers, in granting the prisoner's request, rules that Dr. Keeley must make known the ingredients of his bichloride of gold compound. The court holds that the cure is not a property right nor trade secret. Inasmuch as it is unprotected by patent, and has been in use more than two years, there is nothing to prevent Dr. Keeley's testifying as to its composition, and the court orders him to do so.—*Columbus Medical Journal.*

ANOTHER "FAITH-CURE" FATALITY.—The coroner at Dayton, O., has held Col. B. F. Mead and his wife to be responsible for the death of their twelve-year-old daughter, who was by them permitted to be treated by "faith-cure" methods while she was suffering from tubercular meningitis.

Margarine has been examined for bacteria and is found to be freer from them than butter. The average in butter was 10,000,000 to 20,000,000 microbes to a gramme; in one extreme case 47,000,000. The average in margarine was 4,000,000 to 6,000,000, and the extreme 11,[illegible]00,000. Cold reduced the margarine microbes from 6,500,000 to 230,200, while it only killed off one-third of those of butter; moreover no pathogenic bacteria were discovered in the imitation.—*New York Sun.*

MEDICAL LEGISLATION IN KANSAS.—The Kansas Medical Practice Bill was defeated chiefly through the influence of the Populists. In the House, according to the *Kansas Medical Journal*, Mr. Winters, a Populist member, said: "We Western people can't support your plug-hat doctors. We've got a lot of old women who are better than any of them." Kansas is certainly a most unfortunate State, if its laws are to be made for it by legislators who are swayed by such arguments as that. The logic of Populism as applied to medicine is certainly consistent with that by which it settles financial and other questions.—*Boston Medical and Surgical Journal.*

ACETANILID HEALS CHANCROIDS IN FROM ONE TO SEVEN DAYS.—Dr. Thomas S. K. Morton is reported as saying in *Philadelphia Polyclinic* that upon "chancroids, the effect of acetanilid is most surprising." He states that all soft venereal sores (chancroids) and inflammations "have uniformly healed in from one to seven days, with a single exception," which one was of a phagedenic nature, and required cauterization with nitric acid before it would heal under the acetanilid. He prescribes a drachm of powdered acetanilid. The patient is to wash several times daily, and then rub in the dry powder. If the sore is beneath the prepuce, leave a quantity of the drug inside, which prevents excoriations by urethral discharges. The drug is entirely wanting in odor.—*Virginia Medical Monthly.*

WHEN A CHILD SHOULD EAT.—A child should have nothing whatever from the adult table before a year and a half at the earliest, preferably not until two years. Solid food should not be allowed until after a year, and then it should be bread, gruels, porridge, and possibly an egg; but these should be prepared for it, and given to it by itself, not at the adults' table. To let a child come to the table is only to teach it to beg for things it should not have. Let it be fed before your meals, so that it shall not be tantalized at seeing you eat when it is hungry.—*Annals of Hygiene.*

Ether is preferred, says the *New York Sun's* reporter of European items of interest, as an anæsthetic in Northern countries and chloroform in the South, although ether tends to cause secretions in the air passages and bronchial trouble. One cause is undoubtedly the difficulty of keeping ether in hot climates. But Dr. Lauder Brunton suggests that the general abstention from meat may be another reason for the successful use of chloroform. He is led to this from the increased number of fatalities under chloroform in Edinburgh since the introduction of American and Australian meats, which has made meat eating more common among all classes in Scotland. —*American Therapist.*

Mr. James Payn pays a neat tribute to the doctor, in a recent issue of the *Cornhill Magazine.* "Upon the whole, and for a 'scratch' companion, I prefer a doctor to a man of any other calling. He may not be very good as a conversationalist, but he is rarely very bad—like a cheroot. He has had a genuine experience of life, and has seen down to the depths of it; a sick man does not attempt to deceive his doctor, or put the best face on his character, as he does with a priest. Moreover, what is very unusual, your doctor knows more about you professionally at all events, than you know about yourself. He does not tell you about it, it is true; not a word of that aneurism you carry about with you, and which will some day kill you in half a minute, but your consciousness that he may possess such knowledge makes him interesting. The best suggestions I have had made to me for plots for my novels have come to me from doctors, to whom I have also had cause to be grateful for many things."—*Gaillard's Medical Monthly.*

A Kansas physician has the following printed at the foot of his bill-head: "A prompt settlement of this bill is requested. If bills are paid monthly a discount of ten per cent is given. Bills not paid promptly will be passed to my attorney for collection. If you pay your physician promptly, he will attend you promptly, night or day, rain or shine, while your slow neighbor waits, as he makes the doctor wait, and while he is waiting, the angels gather him in."—*General Practitioner.*

According to statistical reports there were in 1893 in America fully 2,000 women practicing medicine in one or other of its forms, and inclusive of 130 homœopathists. The majority were ordinary practitioners, but among the remainder were 70 hospital physicians or surgeons; 95 professors in the schools; 610 specialists for the diseases of women; 70 alienists; 65 orthopedists; 40 oculists and aurists; and finally, 30 electro-therapeutists. In Canada there is but one medical school exclusively devoted to the training of medical ladies, but in the United States in 1893 there were ten, one of them being a homœopathic establishment.—*Gaillard's Medical Monthly.*

Messrs. P. Blakiston, Son & Co., of Philadelphia, announce the early publication of an authorized translation by Dr. Albert B. Hale, of Chicago, of a "Handbook of Diseases of the Eye," by Dr. A. Eugene Fick, of the University of Zurich. This is one of the most complete, thorough and compact of text-books. Among its other merits it contains a number of very handsome colored illustrations, not of rare or unusual cases, but of practical matters that will greatly aid the student and be of much service to the practitioner. The retail price will be from $3 to $4.

The heat of Calcutta has been excessive. Out of doors in the middle of the day the shade temperature has been as high as 115°. In many of the offices, notwithstanding all the appliances to cool the atmosphere, a temperature as high as 107° has been recorded, and in the coolest part of the city a temperature of 101° has been the average. Many of the judges have been obliged to adjourn their courts, and commercial business has been practically at a standstill. This intense heat has had a disastrous influence on the health of Calcutta, and the death rate has risen as high as 58 per 1,000.—*Northwestern Lancet.*

